# Image Alignment Based on Deep Learning to Extract Deep Feature Information from Images

**DOI:** 10.3390/s25154628

**Published:** 2025-07-26

**Authors:** Lin Zhu, Yuxing Mao, Jianyu Pan

**Affiliations:** State Key Laboratory of Power Transmission Equipment Technology, School of Electrical Engineering, Chongqing University, Chongqing 400044, China

**Keywords:** image alignment, deep learning, feature extraction, infrared and visible images

## Abstract

To overcome the limitations of traditional image alignment methods in capturing deep semantic features, a deep feature information image alignment network (DFA-Net) is proposed. This network aims to enhance image alignment performance through multi-level feature learning. DFA-Net is based on the deep residual architecture and introduces spatial pyramid pooling to achieve cross-scalar feature fusion, effectively enhancing the feature’s adaptability to scale. A feature enhancement module based on the self-attention mechanism is designed, with key features that exhibit geometric invariance and high discriminative power, achieved through a dynamic weight allocation strategy. This improves the network’s robustness to multimodal image deformation. Experiments on two public datasets, MSRS and RoadScene, show that the method performs well in terms of alignment accuracy, with the RMSE metrics being reduced by 0.661 and 0.473, and the SSIM, MI, and NCC improved by 0.155, 0.163, and 0.211; and 0.108, 0.226, and 0.114, respectively, compared with the benchmark model. The visualization results validate the significant improvement in the features’ visual quality and confirm the method’s advantages in terms of stability and discriminative properties of deep feature extraction.

## 1. Introduction

The core objective of image alignment is to establish a spatial mapping relationship between multiple similar scene images captured by different sensors and from various perspectives, thereby achieving alignment of the same target across heterogeneous images [1]. As an important preprocessing step in computer vision, image alignment technology plays a key role in advanced visual tasks such as multimodal image fusion, object detection and tracking, and 3D reconstruction. High-quality alignment results ensure the precise fusion of features in the image space, thereby significantly improving the performance of subsequent multimodal visual tasks.

Heterogeneous image alignment is a challenging research task in the field of computer vision. Due to various factors, such as shooting conditions, shooting angles, and sensor resolution, significant differences in brightness and contrast, geometric distortion, scale inconsistencies, and noise interference often occur between images, making it challenging to achieve ideal results through direct alignment [2]. Among the many detection technologies, infrared thermal imaging and visible light imaging are the two mainstream technologies that have been widely used in many fields such as military, medical, and electric power.

Infrared images are based on thermal radiation imaging of targets, enabling effective detection and identification of scene objects. However, they typically have low resolution and lack detailed texture information. Visible light images align with human visual habits, offering high spatial resolution and clear texture details. Nevertheless, their imaging is easily affected by environmental lighting conditions [3]. Therefore, single infrared or visible light images cannot meet the needs of practical applications, and it is necessary to combine infrared and visible light images to achieve complementary information. Therefore, researching efficient and high-precision image alignment technology is an important prerequisite for achieving deep integration of infrared and visible light image information [4]. The alignment of infrared and visible light images aims to solve the problem of spatial inconsistency caused by rotation, translation, and deformation between images acquired by different sensors. Its core is used to derive a spatial transformation model between them by establishing a correspondence between multimodal image feature points or feature regions [5].

Traditional feature point-based alignment methods rely on multiple iterations of optimization, which can achieve high alignment accuracy, but mainly extract low-level features and lack the ability to capture middle- and high-level semantic information. Although, some feature-based methods have achieved good alignment accuracy, such as RIFT and DASC. However, RIFT is sensitive to nonlinear radiometric differences, and the feature matching performance decreases significantly during extreme illumination changes (e.g., nighttime infrared–visible alignment). DASC has a high memory occupancy, which leads to a decrease in the alignment efficiency, and they are only applicable to the processing of small-sample data. When faced with scenes or platforms with large data volume and high timeliness requirements, low-level features are not sensitive to changes in image illumination, rotation, texture, etc., and the complexity of data processing is extremely high, which limits the alignment effect. However, CrossRAFT has limited generalization ability on large-scale datasets; SuperFusion is very sensitive to noise, resulting in performance degradation at low signal-to-noise ratios; UMF convergence is unstable and only aligned by global features; and SI-BMFE extracts according to a fixed scale structure, making it difficult to deal with multi-scale targets. Therefore, in this paper, we address the above problems and conduct research in terms of extracting high-level features in images, establishing global and local multi-scale feature fusion, and enhancing key features.

This paper makes three main contributions:

1. It proposes an image alignment method based on deep learning to extract deep feature information from images, aiming to fully leverage the global information in both infrared and visible light images to extract key features in the middle and upper layers effectively.
2. It designs a spatial information fusion module (SFM) that integrates spatial pyramid pooling technology to extract deep key features with high stability and strong discriminative power.
3. A feature weight adaptive adjustment mechanism is designed to dynamically adjust weight coefficients based on the stability and discriminative power of features, thereby highlighting the representational capabilities of key features.

The remainder of this paper is organized as follows: We describe the relevant background knowledge in Section 2 and provide a detailed introduction to our alignment network in Section 3. In Section 4, we conduct experiments on two general datasets and discuss the experimental results. This paper concludes with a summary in Section 5.

## 2. Related Work

Currently, image alignment algorithms are mainly divided into three categories: region-based, feature-based, and deep learning-based.

### 2.1. Region-Based Image Alignment Method

The region-based alignment algorithm mainly relies on the spatial features of the reference image to solve the optimal geometric transformation parameters of the image to be matched by optimizing the correlation index. The key is to establish an effective similarity evaluation system and conduct an in-depth analysis of the image gray-scale features to quantify the similarity between the target image and the reference image, thereby achieving pixel-level accurate matching [6]. In the actual implementation process, it is necessary to pre-construct a transformation model and use advanced statistical measures such as normalized cross-correlation (NCC) and mutual information (MI) as evaluation criteria for similarity metrics [7].

Although region-based alignment methods have obvious advantages in theory, their practical application still faces many challenges. First, the algorithm requires high integrity of image pixel information, and noise and geometric distortion can significantly affect the alignment accuracy. Second, because it requires processing of all image pixels, this type of method usually has high computational complexity, which is particularly disadvantageous in application scenarios with high real-time requirements. Therefore, while this method holds an important position in theoretical research, it still has significant limitations in practical engineering applications, particularly in scenarios where computational resources and time costs are constrained, resulting in relatively low application frequency.

### 2.2. Feature-Based Image Alignment Method

Feature-based alignment methods are widely used in alignment tasks due to their excellent image representation capabilities. These methods do not rely on global image information, but instead construct feature matching relationships by extracting salient local features in images, such as geometric elements like points, lines, and surfaces, to derive geometric space transformation models. In the development of feature-based image alignment technology, David Lowe [8] proposed the scale-invariant feature transform (SIFT) algorithm, which laid the theoretical foundation for key point matching. This method detects stable feature points by constructing a Gaussian difference space and has significant noise resistance and scale-invariance characteristics. However, it has high computational complexity and is prone to feature loss in low-texture scenes. To improve computational efficiency, Bay [9] developed the speeded up robust features (SURF) algorithm, which uses integral images and Hessian matrix detection strategies to optimize computational speed while maintaining scale invariance significantly. Rublee [10] further integrated the FAST detector and BRIEF descriptor to propose an ORB algorithm, which achieves both rotation and scale invariance through the construction of image pyramids and direction parameter design.

Traditional feature-based registration methods typically rely on multiple iterations of optimization. This can achieve high accuracy, but the features extracted are mostly low-level features that lack the capture of medium- and high-level semantic information and are only applicable to small sample data processing. When faced with large data volumes and scenarios or platforms with high timeliness requirements, low-level features are not very sensitive to changes in image lighting, rotation, and texture, and the complexity of data processing is extremely high, which limits the alignment effect.

### 2.3. Deep Learning-Based Image Alignment Method

In recent years, the rapid development of deep learning technology has drawn significant attention from the academic community due to its powerful capabilities in feature learning, particularly in its widespread application in tasks such as image classification, semantic analysis, and object detection, demonstrating its immense potential [11]. Unlike traditional registration methods that rely on complex mixed model optimization, deep learning-based registration techniques automatically extract feature points and construct descriptors using neural networks while employing loss functions for supervised learning. This enables more precise acquisition of matching point pairs, significantly improving registration accuracy. In this field, numerous studies have achieved significant progress. For example, Mao [12] introduced a semi-supervised transfer learning strategy by constructing a dual-branch convolutional encoding architecture to optimize the image reconstruction process for the visible spectrum and infrared bands, thereby effectively improving the accuracy of matching. Tang [13] proposed a bidirectional deformation field estimation network that corrects geometric distortions in input images under the supervision of photometric and endpoint constraints. Although it combines semantic perception and bidirectional registration, its feature fusion module is sensitive to noise, resulting in decreased performance in low signal-to-noise ratio scenarios. Furthermore, the end-to-end training of this method relies on a large amount of labeled data, limiting its application in unsupervised or weakly supervised tasks. Although CrossRAFT performs well in multimodal registration, it has high computational complexity and limited generalization ability on large-scale datasets. This method primarily relies on optical flow estimation, resulting in a significant decline in registration accuracy under extreme lighting changes or in low-texture regions. Qiu [14] introduced a robust adaptive variational attention method, which addresses the challenge of precise image matching through a weak boundary constraint strategy. Additionally, D2-Net [15], as an image-based matching algorithm, directly processes raw images to generate feature maps and extracts robust features with high-level semantic information based on delayed feature detection. MAP-Net [16] uses aggregation pooling technology to enhance robustness to geometric distortion and radiation changes in multimodal images. Jiang [17] further combines multi-scale feature extraction and semantic segmentation to propose a multimodal image alignment method suitable for complex backgrounds, using a classic convolutional network to capture robust feature descriptors.

Although these methods perform well in feature extraction and matching, deep learning models are typically accompanied by a significant increase in parameter numbers and computational complexity. In addition, due to the low resolution and blurred details of infrared images, deep learning architectures still face challenges in extracting fine features such as key points, resulting in insufficient feature extraction.

In summary, in order to solve the problem where traditional alignment methods often have difficulty fully extracting deep feature information when processing multimodal images, resulting in limited alignment effects, this paper proposes an infrared and visible light image alignment network for deep feature information extraction.

## 3. Methods

**Figure 1:**
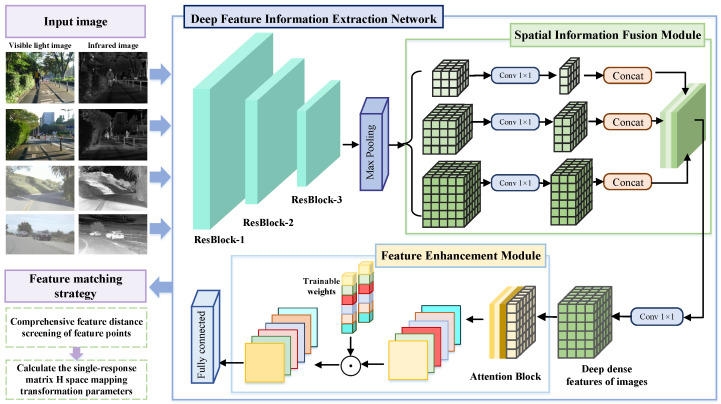
DFA-Net network structure.

This paper proposes a deep feature information extraction for infrared and visible image alignment network (DFA-Net). DFA-Net is based on the ResNet architecture, which is optimized structurally and incorporates a spatial information fusion module (SFM) to embed feature representations containing high-level semantic information, enabling robust extraction and description of multi-scale features. In addition, a feature enhancement module (FEM) based on a self-attention mechanism is designed to perform weighted screening of the deep dense features output by the SFM, highlighting key features with invariance, distinguishability, and repeatability to adapt to the needs of image alignment tasks. Finally, misaligned points are removed using similarity metrics and the RANSAC algorithm, and a geometric transformation model is fitted to further improve matching accuracy. The overall network structure of DFA-Net is shown in Figure 1, and the design and implementation of each module will be detailed below.

### 3.1. Deep Feature Information Extraction Network

**Figure 2:**
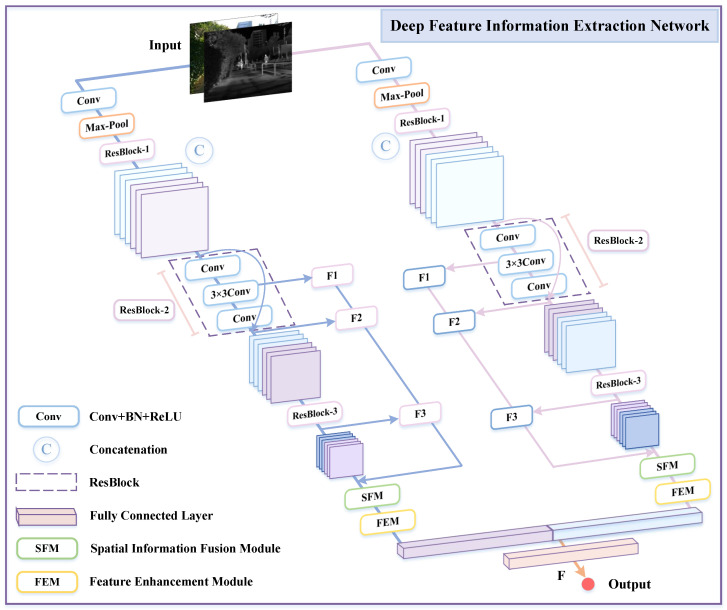
Structure of the deep feature information extraction network.

Neural networks can efficiently learn various features of images through a large number of samples. The descriptors of feature points are typically represented by the outputs of specific layers in a convolutional neural network (CNN), thereby significantly simplifying the complex feature extraction process. ResNet, as a typical representative of CNN, has demonstrated outstanding performance in image feature extraction, classification, and object recognition tasks [18]. Although ResNet-50 and ResNet-101 are widely adopted in research, ResNet-101 is limited in practical applications due to its high computational complexity and time cost. Therefore, this paper selects the moderately deep ResNet-50 as the core network of the deep feature extraction module and optimizes its structure, proposing a deep feature information extraction network (DFEN), as shown in Figure 2. Specifically, this module uses only three residual blocks to reduce the computational complexity of the network. After the ResBlock-3 output, an SFM based on spatial pyramid pooling is introduced. Regardless of changes in the input image size, the SFM generates fixed-size outputs, significantly enhancing the scale invariance of the image and effectively reducing the risk of overfitting.

Input images of any size and their corresponding reference images into the deep feature extraction network at their original resolution. In the input image, each 8 × 8 pixel region is defined as a feature point, and multi-scale feature vectors are obtained by extracting the 8 × 8, 16 × 16, and 32 × 32 pixel regions surrounding the feature points. These feature vectors correspond to the outputs of ResBlock-1, ResBlock-2, and ResBlock-3 in the DFEN. This paper discards the low-level features extracted by ResBlock-1, uses the hidden layer output of ResBlock-2 to obtain the first feature vector $F_{1}$, and combines the outputs of ResBlock-2 and ResBlock-3 to construct the feature description vector for registration.

Assuming the input image size is N × N, this paper uses the output of the second convolutional layer in ResBlock-2 as the first feature vector $F_{1}$. The size of $F_{1}$ is (N/8) × (N/8) × 512, meaning that each 8 × 8 pixel region in the input image corresponds to a 512-dimensional vector in $F_{1}$. For each 16 × 16 pixel region in the input image, it corresponds to a 512-dimensional vector in the ResBlock-2 output, denoted as $R_{block-2}$, with a size of (N/16) × (N/16) × 512. Since a single feature vector in ResBlock-2 is shared by four defined feature points, the second feature map is generated using $R_{block-2}$, thereby obtaining the second feature vector $F_{2}$ of the input image.

Additionally, each 32 × 32 pixel region in the input image generates a 1024-dimensional feature vector, meaning that 16 feature points share a single 1024-dimensional feature vector, corresponding to the 1024-dimensional vector in the ResBlock-3 output, denoted as $R_{block-3}$, with dimensions (N/32) × (N/32) × 1024. Since each feature vector in $R_{block-3}$ is shared by 16 defined feature points, a Kronecker product is performed on the output of $R_{block-3}$ to obtain the third feature vector $F_{3}$.

(1) $$F_{2}=R_{block-2}⊗E_{2\times 2\times 1}$$

(2) $$F_{3}=R_{block-3}⊗E_{4\times 4\times 1}$$

Among them, ⊗ is defined as the Kronecker product, $E_{2\times 2\times 1}$ is defined as a 2 × 2 × 1 tensor filled with 1, and $E_{4\times 4\times 1}$ is defined as a 4 × 4 × 1 tensor filled with 1. Compared with other feature expansion methods, the Kronecker product enables full interaction between feature vectors, revealing their cross-relationships, which is crucial for multimodal feature fusion. Additionally, the Kronecker product maintains precise dimensions, whereas the outer product generates redundant dimensions. Therefore, after a comprehensive comparison, this paper selects the Kronecker product for feature vector calculation. Define $A=a_{ij}\in c^{m\times n}$ and $B=b_{ij}\in c^{p\times q}$, where the Kronecker product of *A* is a block matrix defined as follows:

(3) $$A⊗B=\left[\begin{array}{cccc} a_{11}B & a_{12}B & \ldots & a_{1n}B\\ a_{21}B & a_{22}B & \ldots & a_{2n}B\\ \vdots & \vdots & \ddots & \vdots \\ a_{m1}B & a_{m2}B & \ldots & a_{mn}B \end{array}\right]\in c^{mp\times nq}$$

Then, the three output feature vectors generated by DFEN, namely $F_{1}$, $F_{2}$, and $F_{3}$, are concatenated into a feature description graph *F*, which contains multi-layer information and has a size of (N/8) × (N/8) × 2048. Each 2048-dimensional component in *F* corresponds to an 8 × 8 pixel region in the input image. After obtaining $F_{1}$, $F_{2}$, and $F_{3}$, the feature map needs to be normalized to unit variance:

(4) $$F_{i}\leftarrow \frac{F_{i}}{\sigma (F_{i})},i=1,2,3$$

where $\sigma (F_{i})$ is the standard deviation of $F_{i}$. The feature vectors of different dimensions for each feature point are generated by $F_{1}$, $F_{2}$, and $F_{3}$, respectively, ultimately forming a feature vector descriptor. This improvement not only overcomes the degradation problem that occurs in deep neural networks but also realizes feature association from low to high levels, enabling the extracted features to have strong generalization performance.

### 3.2. Multi-Scale Adaptive Spatial Information Fusion Structure

**Figure 3:**
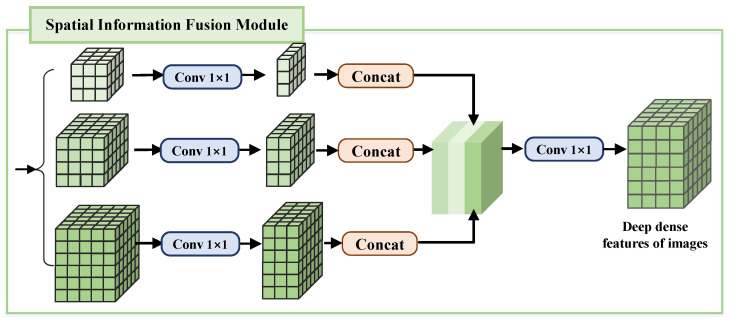
Structure of the spatial information fusion module.

By integrating spatial context information, the SFM enables the network to effectively simulate complex geometric distortions between infrared and visible light images, which is crucial for the task of aligning infrared and visible light images. As shown in Figure 3, the SFM aggregates spatial context information based on different local regions and combines multi-scale pooling and convolution operations [19], thereby significantly improving the performance of the DFA-Net network in mining and integrating spatial structure information.

The extracted features of different scales are cascaded and fed into a spatial information fusion module containing a three-layer feature pyramid. This module adopts a multi-scale pooling kernel design, achieving feature extraction from different subregions through a simplified operation process. Therefore, it is necessary to reasonably set the size of the pooling kernels between adjacent layers. In this paper, the pooling operators in the SFM are set to 1 × 1, 2 × 2, and 4 × 4, and pooling operations are performed on the input feature maps to generate three feature maps of different scales, with sizes being one-quarter, one-half, and the original size of the input feature map, respectively. To preserve the significance of global features, a 1 × 1 convolution layer is introduced after each pyramid level to reduce the feature size to one-third of the original size. Next, the dimensionless multi-scale feature maps are upsampled using different scaling factors to restore them to the same size as the input feature maps and then concatenated along the channel dimension. Finally, the concatenated feature maps are fused along the channel dimension using a 1 × 1 convolution kernel, thereby aggregating multi-scale spatial context information.

It is worth noting that the core of the image matching task lies in identifying and locating key features that are highly representative and discriminative, which requires more attention to local information. In order to reduce the interference of mismatched areas on the matching results, it is usually necessary to limit the search range when matching corresponding points. Therefore, the SFM does not use global pooling operations but instead chooses the maximum pooling strategy to retain the significant geometric structural features in infrared and visible light images while avoiding the weakening of feature information.

### 3.3. Feature Enhancement Structure Based on Self-Attention Mechanism

When dealing with infrared and visible light images with complex backgrounds, research usually focuses on the alignment effect of foreground objects. However, complex background areas often contain a large number of feature points, which can significantly interfere with the alignment results [20]. Therefore, during the feature point selection process, it is necessary to prioritize the retention of key feature points to ensure that the alignment results mainly rely on the feature information on the target object [21].

**Figure 4:**
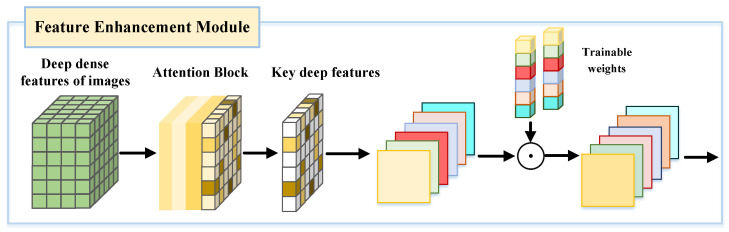
Structure of the feature enhancement module.

The SFM can output dense features containing multi-scale semantic information, but the contribution of these features to the matching task varies significantly. Some features are located in homogeneous regions with poor texture, exhibiting poor representativeness and robustness, and are therefore unsuitable as candidate features for image matching. To address this issue, this paper introduces the FEM after the SFM, with the FEM network structure being shown in Figure 4. This module uses a self-attention mechanism to weight features. The FEM module uses multi-head attention (head = 4) with a total hidden dimension of 512. During training, the FEM can assign higher attention weights to important features, thereby highlighting key features and suppressing redundant information, effectively reducing noise and error interference and improving the accuracy of feature selection.

The FEM maps the overall feature vectors of the input image and then analyzes the intrinsic dependencies between feature vector sequences to effectively extract and integrate global information. The deep dense features of the image output by the SFM are used as input for the FEM, where the input is represented as $F=(F_{1},F_{2},\ldots ,F_{i})$, with $F_{i}$ being the *i*-th feature vector. Through the learnable weight matrices $W_{Q}$, $W_{K}$, and $W_{V}$, the input sequence is mapped to query, key, and value vectors, respectively:

(5) $$Q=FW_{Q}$$

(6) $$K=FW_{K}$$

(7) $$V=FW_{V}$$

By calculating the dot product of the query vector *Q* and the key vector *K*, we obtain the attention score matrix, which represents the relevance of each element in the sequence to the other elements. The association strength of key deep features is expressed as:

(8) $$\mathrm{Attention}\;\mathrm{Scores}=\frac{QK^{T}}{\sqrt{d_{k}}}$$

where $d_{k}$ is the dimension of the key vector, which regulates the magnitude of the dot product, effectively avoiding gradient vanishing or gradient explosion. The attention score matrix is normalized using softmax, resulting in the attention weight matrix:

(9) $$\mathrm{Attention}\;\mathrm{Weights}=\mathrm{softmax}(\frac{QK^{T}}{\sqrt{d_{k}}})$$

The final output is obtained by performing a weighted sum of the value vector *V* using the trainable attention weights:

(10) Output=AttentionWeights·V

In the process of calculating attention weight scores, the input features are first normalized to ensure that the distribution of weight scores is within a reasonable numerical range. Once the attention module has completed training, the network uses the calculated weighted scores to select features that are highly relevant to the deep information in the image.

### 3.4. Feature Matching Strategy

During the feature matching process, the feature description vectors must first be normalized. This paper uses Euclidean distance as a similarity evaluation index and calculates the spatial distance between feature descriptors to achieve feature point matching. For feature points on the alignment subject, considering that each point contains feature vectors of different dimensions, this paper uses comprehensive feature distance as the basis for measuring the similarity of multimodal image feature points. Given the differences in the dimensions of each feature vector, a weight adjustment mechanism is introduced for compensation. The formula for defining comprehensive feature distance is:

(11) $$d_{all}=d_{F_{1}}+d_{F_{2}}+d_{F_{3}}$$

(12) $$d_{F_{1}}=2d_{512}\left(a_{i},b_{j}\right)$$

(13) $$d_{F_{2}}=\sqrt{2}d_{512}\left(a_{i},b_{j}\right)$$

(14) $$d_{F_{3}}=d_{1024}\left(a_{i},b_{j}\right)$$

where $d_{all}$ denotes the comprehensive feature distance; $d_{F_{1}}$, $d_{F_{2}}$, and $d_{F_{3}}$ denote the feature distances of the output feature vectors $F_{1}$, $F_{2}$, and $F_{3}$, respectively; $a_{i}$ and $b_{j}$ denote the two nearest neighbour feature matching points of the infrared and visible light images; $d_{512}\left(a_{i},b_{j}\right)$ denotes the Euclidean distance between the 512-dimensional vectors of the two feature points; and $d_{1024}\left(a_{i},b_{j}\right)$ denotes the Euclidean distance between the 1024-dimensional vectors of the two feature points.

When the ratio of the comprehensive feature distance between a feature point and its nearest neighbour to the comprehensive feature distance between the second nearest neighbour is less than a predefined threshold, the matching relationship between the feature point and its nearest neighbour is deemed valid. After the initial matching of feature points is completed, the classic RANSAC algorithm is used to remove mismatched point pairs, leaving only correctly matched feature point pairs.

After completing the feature point pair selection, an affine transformation matrix is generated using the effective matching points. By establishing a correspondence mapping relationship between the feature vectors of infrared and visible light image regions, the rotation and scale differences between images are addressed. Since the mapping relationships between different feature point pairs vary, global optimization is required to determine the optimal transformation parameters. Specifically, the RANSAC algorithm randomly selects matching point pairs to construct a homographic model, calculates the projection errors of all feature point pairs, and sets a maximum allowable threshold. Points with errors below the threshold are included in the optimal set. If the current set is better than the optimal set, the current set is set as the optimal set. Through iterative optimization, the optimal transformation model with the minimum projection error is ultimately obtained. The formula for calculating the iteration count *k* is:

(15) $$k=\frac{\log\left(1-z\right)}{\log\left(1-w^{n}\right)}$$

where *z* is the confidence level, *w* is the proportion of the optimal set in the dataset, and *n* is the minimum number of samples required for iterative selection. Then, the filtered points are used to generate the homography matrix *H* required for image transformation, where the formula for *H* is:

(16) $$H=\left(\begin{array}{ccc} h_{11} & h_{12} & h_{13}\\ h_{21} & h_{22} & h_{23}\\ h_{31} & h_{32} & 1 \end{array}\right)$$

For feature point pairs on the image, the coordinates can be calculated using the following formula:

(17) $$\left(\begin{array}{c} x^{\prime }\\ y^{\prime }\\ 1 \end{array}\right)=\left(\begin{array}{ccc} h_{11} & h_{12} & h_{13}\\ h_{21} & h_{22} & h_{23}\\ h_{31} & h_{32} & 1 \end{array}\right)\left(\begin{array}{c} x\\ y\\ 1 \end{array}\right)$$

where (x,y) is the position coordinate of the feature point before transformation and $\left(x^{\prime },y^{\prime }\right)$ is the position coordinate of the feature point after transformation. After calculating the homography matrix, matrix multiplication is performed with the homogeneous coordinates of the corresponding points in the reference image to obtain the spatial mapping relationship between the two images, thereby completing the alignment of the infrared and visible light images.

## 4. Experiments and Discussion

### 4.1. Image Dataset and Evaluation Metrics

This paper uses the MSRS dataset [22] and RoadScene dataset [23], which are widely recognized in the field of infrared and visible light image alignment. The MSRS dataset contains 1444 pairs of infrared and visible light images with a resolution of 480 × 640, while the RoadScene dataset is selected from the FLIR dataset and includes 221 pairs of images. The image pairs in these datasets cover a variety of typical scenes, including roads, pedestrians, and vehicles, and exhibit high representativeness and diversity. The original datasets were divided into a training set (80%), a validation set (10%), and a test set (10%). During the data preprocessing stage, missing values were first handled. For numerical features, median imputation was used. For categorical features, mode imputation was used. Then, a normalization method was applied to scale the range to [0, 1].

The experiment was implemented using the PyTorch 2.1.0 framework and run on an NVIDIA RTX3090 (NVIDIA, Shenzhen, China) hardware platform. The experimental hyperparameter settings are shown in Table 1 below. The initial learning rate was set to $10^{-3}$, and the training process underwent a total of 1100 iterations. To comprehensively evaluate the performance of DFA-Net, this paper analyzes it from both qualitative and quantitative perspectives and compares it with advanced methods such as RIFT [24], DASC [25], CrossRAFT [26], SuperFusion [13], UMF [27], and SI-BMFE [28]. In the quantitative evaluation, four metrics were employed: root mean square error (RMSE), structure similarity index measure (SSIM), mutual information (MI), and normalized cross-correlation (NCC), to comprehensively assess the quality of the registration results.

### 4.2. Ablation Experiment

**Figure 5:**
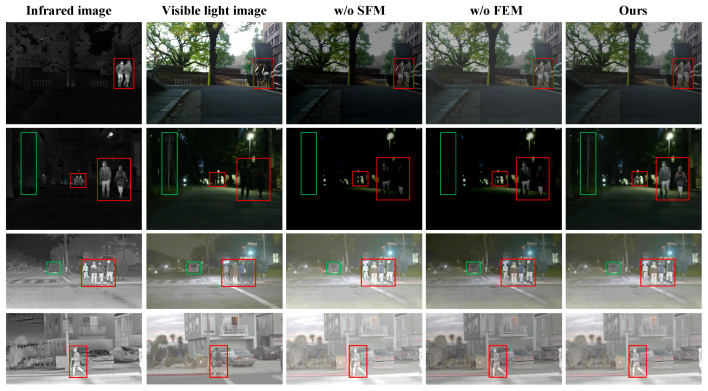
Visualization of the ablation experiment results. Red boxes indicate people. Green boxes indicate objects such as streetlights, vehicles, etc.

To assess the actual contribution of each module in the network to the alignment task, this section conducts ablation experiments to analyze the impact of the SFM and FEM on model performance. The experiments are based on the classic deep residual network ResNet-50 as the base model, systematically comparing the optimization effects of these two modules on DFA-Net performance by introducing or removing the SFM and FEM, respectively. The experiments were conducted on the MSRS dataset and the RoadScene dataset, with the specific results being shown in Table 2 and Table 3. The visualization results of the ablation experiment are shown in Figure 5. By progressively replacing or integrating the proposed modules into the original ResNet-50 model, eight sets of comparative experimental data were obtained. The experimental results demonstrate the specific impact of different module combinations on alignment performance.

#### 4.2.1. Analysis of Ablation Experiments on the SFM

As shown in Table 2, the experimental results in the MSRS dataset indicate that the introduction of the SFM significantly improves performance metrics. Comparing the results in rows 1 and 2, it can be seen that introducing the SFM into the base model reduces RMSE by 0.209 and improves SSIM, MI, and NCC by 0.039, 0.038, and 0.084, respectively. Comparing the results in rows 3 and 4, compared with adding only the FEM, RMSE decreased by 0.263, and the overall performance metrics of SSIM, MI, and NCC improved by 0.042, 0.04, and 0.09, respectively.

The experimental results in the RoadScene dataset are shown in Table 3. From the comparison results in rows 1 and 2, it can be seen that introducing the SFM into the base model reduced RMSE by 0.149, and SSIM, MI, and NCC improved by 0.029, 0.036, and 0.046, respectively. In the comparison between rows 3 and 4, compared with adding only the FEM, RMSE decreased by 0.151, and the overall performance metrics of SSIM, MI, and NCC improved by 0.035, 0.037, and 0.047, respectively.

These data fully prove that the introduction of the SFM enables the DFA-Net network to effectively retain significant geometric structure information in infrared and visible light images during feature extraction, fully expressing the deep features and detailed information of the image. It performs particularly well in terms of image brightness, contrast, and structural similarity, avoiding the degradation of feature information in the source image and significantly enhancing the network’s alignment capabilities. In addition, by comparing the ablation experiment data of the SFM in the two datasets, it can be seen that the SFM shows better performance indicators in the MSRS dataset. This is because the images in the MSRS dataset show richer image texture information than those in the RoadScene dataset, so the SFM can fully mine the deep information of the image and improve the accuracy of infrared and visible light image alignment.

#### 4.2.2. Analysis of Ablation Experiments on the FEM

As shown in Table 2, the experimental results in the MSRS dataset indicate that, when comparing the first and third rows, introducing the FEM into the base model reduces RMSE by 0.398, while SSIM, MI, and NCC performance improves by 0.113, 0.123, and 0.121, respectively. In the comparison between the second and fourth rows, compared to adding only the SFM, RMSE decreased by 0.452, and the overall performance metrics of SSIM, MI, and NCC improved by 0.116, 0.125, and 0.127, respectively.

The experimental results in the RoadScene dataset are shown in Table 3. Comparing the first and third rows, introducing the FEM into the base model reduced RMSE by 0.322, and improved the overall performance metrics of SSIM, MI, NCC improved by 0.073, 0.189, and 0.067, respectively. In the comparison between the second and fourth rows, compared to adding only the SFM, RMSE decreased by 0.324, and the overall performance metrics of SSIM, MI, and NCC improved by 0.079, 0.190, and 0.068, respectively.

Ablation experiment data indicate that the FEM assigns higher weights to important deep features through the attention mechanism, thereby suppressing the influence of non-significant features, strengthening the expression of key features, and effectively improving the registration performance of the DFA-Net network. In addition, compared with the SFM, the FEM has a more significant effect on improving alignment accuracy. This is mainly attributed to the attention mechanism, which can more accurately screen and effectively utilize deep features that can characterize the texture information of key areas, enhance the expression of features suitable for image matching, and enable the deep feature information of image pairs to be distributed sparsely and uniformly, which is more conducive to the calculation and optimization of image transformation parameters.

### 4.3. Comparison of Results with Other Methods

This paper compares the proposed DFA-Net network model with six other advanced image alignment algorithms on the MSRS and RoadScene datasets, including RIFT [24], DASC [25], CrossRAFT [26], SuperFusion [13], UMF [27], and SI-BMFE [28]. Twenty pairs of infrared and visible light images were selected from each of the MSRS and RoadScene datasets to form the test sets.

**Figure 6:**
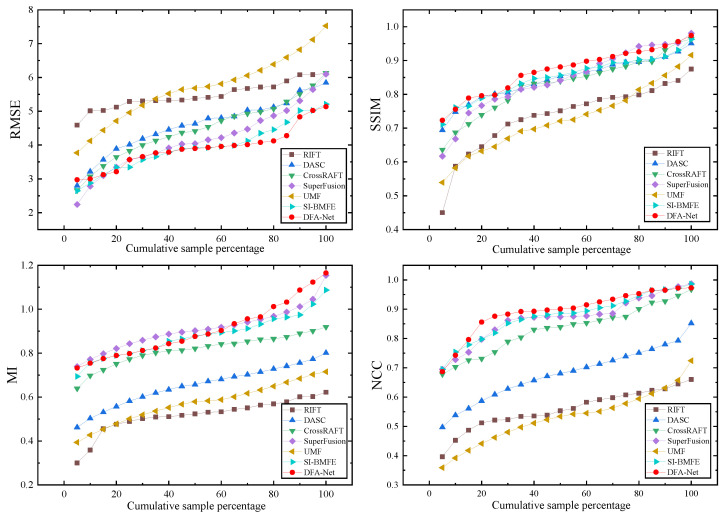
Quantitative comparison with SOTA methods in the MSRS dataset.

**Figure 7:**
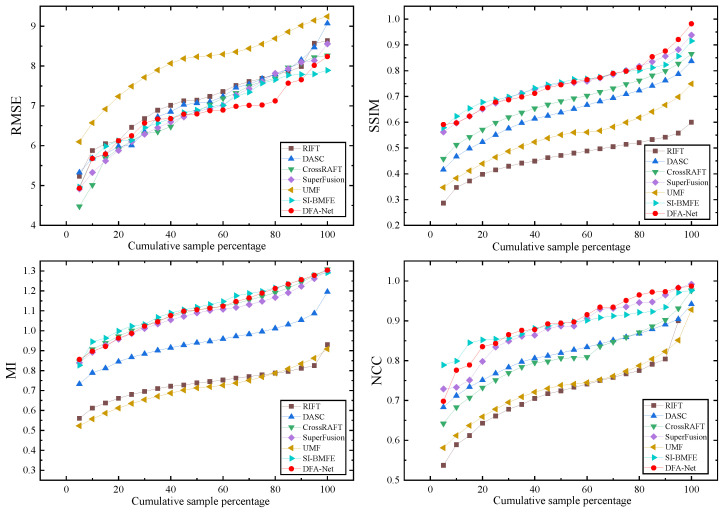
Quantitative comparison with SOTA methods in the RoadScene dataset.

As shown in Table 4 and Table 5, this paper compares DFA-Net with six mainstream methods on the MSRS and RoadScene datasets. The best results are marked in red, and the second-best results are marked in blue. In terms of quantitative analysis, the RMSE, SSIM, MI, and NCC values of DFA-Net on the two datasets are 3.910, 0.871, 0.906, and 0.894; and 6.782, 0.752, 1.099, and 0.893, respectively. Figure 6 and Figure 7 detail the distribution of the four metrics across methods as the sample proportion increases.

According to the analysis of experimental data, DFA-Net achieved good alignment results, with the aligned images performing well in terms of structural preservation, brightness consistency, and contrast matching. Although SuperFusion and SI-BMFE are slightly better than DFA-Net in terms of the MI index, the difference is only 0.004 and 0.011, respectively, which is mainly attributed to the fact that SuperFusion and SI-BMFE use segmentation networks to balance the deviation of the input images, thereby improving the similarity of image alignment. RIFT performs feature point detection based on the principle of phase consistency. This is sensitive to large-scale viewpoint changes but still has shortcomings in terms of image structure similarity preservation and alignment accuracy optimization. DASC uses continuous Gaussian smoothing approximation to determine the relative scale, which is only effective within a limited range of scale changes and is not suitable for mining deep feature information in images. Due to its high computational complexity, alignment takes significantly longer than other methods. Although CrossRAFT proposes a new matching mechanism for cross-modal flow estimation, its alignment performance in complex scenes in the MSRS dataset still lags behind that of this method. The performance of UMF is relatively poor, mainly because UMF focuses on joint alignment and uses the displacement vector field between the predicted distortion and the pseudo-infrared image to reconstruct the aligned infrared image, which does not adequately represent the deep information in the infrared and visible light images.

Table 4 and Table 5 show the running times of each method to evaluate the computational speed of image alignment methods. The experimental data show that deep learning-based models generally outperform traditional algorithms, namely RIFT and DASC, in terms of time performance metrics. This is mainly due to the acceleration effect brought about by the parallel computing architecture of GPUs. The DFA-Net algorithm integrates multi-scale spatial pyramid pooling with an adaptive, optimized attention mechanism, effectively enhancing the accuracy of deep feature representation and the robustness of the model. However, while this improvement enhances model performance, it also introduces additional computational overhead, resulting in slightly lower computational efficiency compared with other state-of-the-art algorithms. Although the network’s performance in terms of time is not optimal, it still maintains a relatively ideal execution speed, demonstrating an effective balance between accuracy and efficiency in algorithm design.

In terms of overall performance comparison, DFA-Net consistently maintains a leading advantage in RMSE, SSIM, and NCC metrics. This is primarily due to the synergistic interaction between the SFM and FEM in the DFA-Net network architecture: the SFM achieves deep extraction of multi-scale features, while the FEM effectively enhances feature expression capabilities through a key feature selection mechanism, thereby significantly improving the network’s registration performance.

**Figure 8:**
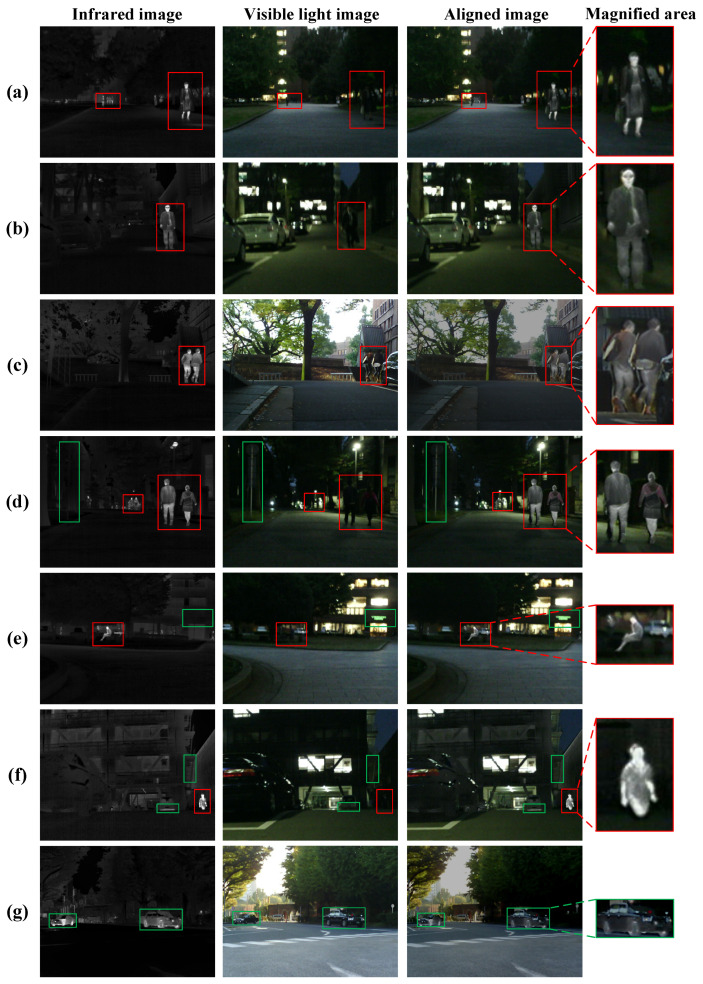
Image alignment visualization results on the MSRS dataset. Red boxes indicate people, green boxes indicate environmental references such as vehicles, streetlights, etc. (**a**–**c**) highlight the spatial alignment effect of pedestrian targets; (**d**–**f**) verify the accurate alignment of multi-level targets in composite scenes of people and backgrounds; and (**g**) shows the alignment effect of vehicle targets.

**Figure 9:**
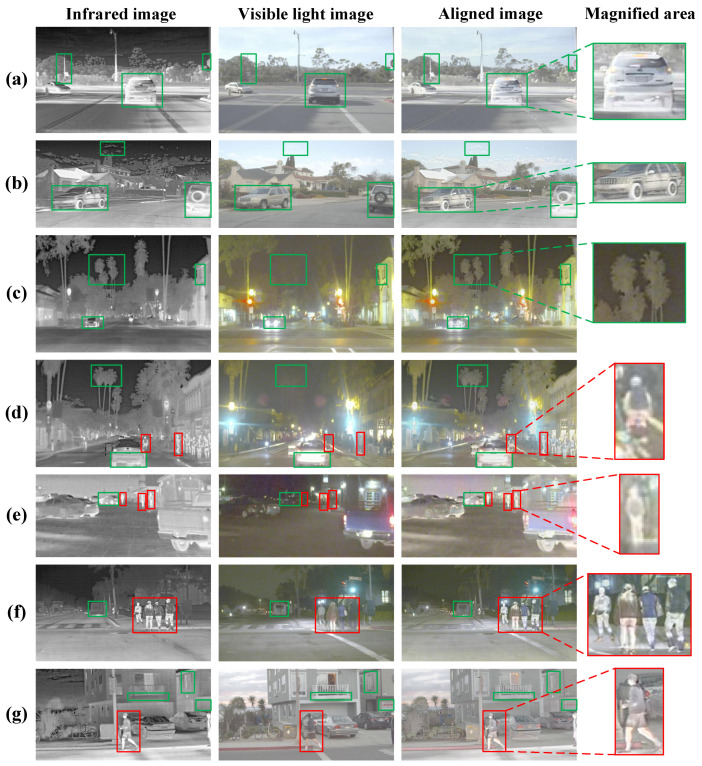
Image alignment visualization results on the RoadScene dataset. Red boxes indicate people, green boxes indicate vehicles, foliage, buildings and other objects. (**a**–**c**) highlight the registration between vehicles and backgrounds; (**d**–**f**) demonstrate the consistency of multi-object registration involving people, vehicles, and backgrounds; and (**g**) shows the registration results for people and backgrounds.

Figure 8 and Figure 9 show the visualization results of infrared and visible light image alignment of DFA-Net in the MSRS and RoadScene datasets, respectively. As shown in Figure 8, (a–c) highlight the spatial alignment effect of pedestrian targets; (d–f) verify the accurate alignment of multi-level targets in composite scenes of people and backgrounds; and (g) shows the alignment effect of vehicle targets. Whether in single-target scenes or complex multi-target environments, DFA-Net can effectively align infrared thermal radiation features and visible light texture details, with natural transitions between target areas in the aligned images and no artefacts or deformation distortions in key areas. As shown in Figure 9, (a–c) highlight the registration between vehicles and backgrounds; (d–f) demonstrate the consistency of multi-object registration involving people, vehicles, and backgrounds; and (g) shows the registration results for people and backgrounds. The experimental results show that even when faced with low-contrast images in the RoadScene dataset, DFA-Net can still accurately identify major targets such as pedestrians and vehicles while retaining detailed features such as leaves and buildings, achieving effective fusion of infrared and visible light image information and obtaining good alignment results, enabling intuitive and accurate expression of target information in infrared and visible light image pairs.

As such, DFA-Net not only performs well in single-object scenarios but also demonstrates outstanding deep feature extraction and registration capabilities in complex multi-object environments and low-contrast conditions, showcasing excellent generalization ability. With a series of studies on low-light image enhancement methods [29,30], this method can be applied to complex environments such as power plants in combination with actual scene registration requirements. However, its adaptability to specific scenes still needs further optimization.

## 5. Conclusions

This paper proposes a deep learning-based image feature information extraction and alignment method (DFA-Net) to solve the problem of traditional alignment methods losing a large amount of high-level feature information in images. Specifically, this paper uses a deep residual network as the basic framework and achieves robust extraction and description of deep features through structural optimization and the introduction of a spatial information fusion module (SFM). A feature enhancement module (FEM) is also designed to perform weighted screening of dense features and highlight key features. Experimental results on the MSRS and RoadScene datasets demonstrate that compared with the baseline model, the RMSE metric is reduced by 0.661 and 0.473, respectively, while the SSIM, MI, and NCC increased by 0.155, 0.163, and 0.211; and 0.108, 0.226, and 0.114, respectively. DFA-Net can effectively integrate global and local contextual information to extract stable and discriminative deep key features, which not only meet the requirements of image alignment tasks but also lay the foundation for its application research.

With the continuous upgrading of image alignment technology, there are still several key issues in this field that urgently need to be addressed. In the future, relevant research can be conducted in the following areas:

1. Optimization of algorithm efficiency: While current deep learning-based registration methods offer advantages in terms of accuracy, their high computational complexity makes it challenging to meet the efficiency constraints of engineering applications. Future research could explore hybrid architecture designs by integrating traditional feature matching methods with deep feature representation techniques to create lightweight models with feature sharing mechanisms, thereby enhancing computational efficiency while maintaining registration accuracy.
2. Task collaboration framework design: Current research mostly treats registration and fusion as independent tasks, leading to redundancy in the feature extraction process. For example, in a power inspection scenario, the future research could construct a joint optimization framework by designing shared feature encoders and adaptive weight allocation mechanisms to achieve collaborative optimization of registration parameter estimation and feature fusion. This end-to-end architecture not only reduces computational resource consumption but also enhances system performance through feature reuse mechanisms, driving the transition of the technology towards engineering applications.

## Figures and Tables

**Table 1:** Experimental hyperparameter settings.

Parameter Name	Optimizer	Batch Size	Epochs	Initial Learning Rate
Parameter settings	Adam	32	1100	$10^{-3}$

**Table 2:** Result of ablation experience in the MSRS dataset.

Baseline	SFM	FEM	RMSE	SSIM	MI	NCC
✓	×	×	4.571	0.716	0.743	0.683
✓	✓	×	4.362	0.755	0.781	0.767
✓	×	✓	4.173	0.829	0.866	0.804
✓	✓	✓	3.910	0.871	0.906	0.894

**Table 3:** Result of ablation experience in the RoadScene dataset.

Baseline	SFM	FEM	RMSE	SSIM	MI	NCC
✓	×	×	7.255	0.644	0.873	0.779
✓	✓	×	7.106	0.673	0.909	0.825
✓	×	✓	6.933	0.717	1.062	0.846
✓	✓	✓	6.782	0.752	1.099	0.893

**Table 4:** Comparision with SOTA methods in the MSRS dataset.

Model	RMSE	SSIM	MI	NCC	Time (s)
RIFT	5.473	0.736	0.517	0.558	9.457
DASC	4.583	0.846	0.653	0.679	26.624
CrossRAFT	4.476	0.830	0.815	0.833	2.641
SuperFusion	4.164	0.845	0.910	0.871	1.094
UMF	5.679	0.728	0.572	0.531	3.931
SI-BMFE	3.944	0.852	0.879	0.878	3.847
DFA-Net	3.910	0.871	0.906	0.894	2.605

**Table 5:** Comparision with SOTA methods in the RoadScene dataset.

Model	RMSE	SSIM	MI	NCC	Time (s)
RIFT	7.141	0.465	0.735	0.714	10.690
DASC	7.061	0.638	0.943	0.818	13.478
CrossRAFT	6.840	0.678	1.091	0.810	2.599
SuperFusion	6.877	0.747	1.080	0.880	1.307
UMF	8.062	0.545	0.712	0.736	3.624
SI-BMFE	6.868	0.748	1.110	0.889	4.347
DFA-Net	6.782	0.752	1.099	0.893	3.235

## Data Availability

MSRS dataset (https://github.com/Linfeng-Tang/MSRS accessed on 27 June 2024) and the RoadScene dataset (https://github.com/hanna-xu/RoadScene accessed on 7 August 2024).

## Abbreviations

The following abbreviations are used in this manuscript:

DFA-Net	Deep feature information image alignment network
DFEN	Deep feature information extraction network
SFM	Spatial information fusion module
FEM	Feature enhancement module
RMSE	Root mean square error
SSIM	Structure similarity index measure
MI	Mutual information
NCC	Normalized cross-correlation
